# Zoom, Zoom, Baby! Assessing Mother-Infant Interaction During the Still Face Paradigm and Infant Language Development *via* a Virtual Visit Procedure

**DOI:** 10.3389/fpsyg.2021.734492

**Published:** 2022-02-16

**Authors:** Nancy L. McElwain, Yannan Hu, Xiaomei Li, Meghan C. Fisher, Jenny C. Baldwin, Jordan M. Bodway

**Affiliations:** ^1^Department of Human Development and Family Studies, College of Agricultural, Consumer, and Environmental Sciences, University of Illinois at Urbana-Champaign, Urbana, IL, United States; ^2^Beckman Institute for Advanced Science and Technology, University of Illinois at Urbana-Champaign, Urbana, IL, United States

**Keywords:** infant stress, language development, mother-infant interaction, virtual visits, participant experience

## Abstract

The COVID-19 pandemic has necessitated innovations in data collection protocols, including use of virtual or remote visits. Although developmental scientists used virtual visits prior to COVID-19, validation of virtual assessments of infant socioemotional and language development are lacking. We aimed to fill this gap by validating a virtual visit protocol that assesses mother and infant behavior during the Still Face Paradigm (SFP) and infant receptive and expressive communication using the Bayley-III Screening Test. Validation was accomplished through comparisons of data (i.e., proportions of missing data for a given task; observed infant and maternal behaviors) collected during in-person laboratory visits and virtual visits conducted *via* Zoom. Of the 119 mother-infant dyads who participated, 73 participated in lab visits only, 13 participated in virtual visits only, and 33 dyads participated in a combination of lab and virtual visits across four time points (3, 6, 9, and 12 months). Maternal perspectives of, and preferences for, virtual visits were also assessed. Proportions of missing data were higher during virtual visits, particularly for assessments of infant receptive communication. Nonetheless, comparisons of virtual and laboratory visits within a given time point (3, 6, or 9 months) indicated that mothers and infants showed similar proportions of facial expressions, vocalizations and directions of gaze during the SFP and infants showed similar and expected patterns of behavioral change across SFP episodes. Infants also demonstrated comparable expressive and receptive communicative abilities across virtual and laboratory assessments. Maternal reports of ease and preference for virtual visits varied by infant age, with mothers of 12-month-old infants reporting, on average, less ease of virtual visits and a preference for in-person visits. Results are discussed in terms of feasibility and validity of virtual visits for assessing infant socioemotional and language development, and broader advantages and disadvantages of virtual visits are also considered.

## Introduction

The COVID-19 pandemic and related restrictions have created challenges for developmental research that relies heavily on traditional in-person methods of data collection. Yet, in meeting those challenges, and building on psychological researchers' successful use of online testing platforms with adults, adolescents, and school-age children (e.g., Buhrmester et al., 2011; Germine et al., 2012; Griffiths et al., 2019), developmental scientists have explored and fine-tuned creative and potentially transformative solutions to conducting research with infants and young children. Although online methods were in use by developmental researchers prior to COVID-19 (e.g., Scott and Schulz, 2017; Tran et al., 2017), the need for such methods during the pandemic has spurred further development and proliferation (see Garrisi et al., 2020; Su and Ceci, 2021). The majority of online or virtual validation studies, to date, have focused on cognitive developmental assessments, whereas validated virtual assessments of infant socioemotional and language development have been sparse. We aimed to fill this gap.

As with almost all facets of life—research and otherwise—across the globe, our longitudinal investigation of infant development was halted in March 2020. We quickly pivoted to a virtual visit protocol using a video conferencing platform, which resulted in a unique opportunity to compare laboratory and virtual visits in assessing infant socioemotional and language functioning. Specifically, we assessed (a) infant and maternal behavior and infant response to stress during the Still Face Paradigm (SFP; Tronick et al., 1978) using a micro-behavioral coding approach, and (b) infant language development using the expressive and receptive communication subtests of the Bayley-III Screening Test (Bayley, 2006a). We also examined maternal perceptions of, and preferences for, virtual vs. laboratory visits.

Our study complements innovative efforts by cognitive developmentalists to collect data *via* online platforms. Asynchronous unmoderated platforms, such as LookIt (Scott and Schulz, 2017; Scott et al., 2017) and Amazon Mechanical Turk (Tran et al., 2017), have been used to conduct infant looking-time studies. Although asynchronous studies with infants and young children have demonstrated feasibility (e.g., Scott and Schulz, 2017; Tran et al., 2017; Rhodes et al., 2020), large portions of data (e.g., 40% in Tran et al., 2017) are typically excluded due to technical problems or procedural errors (e.g., infant position in the video). Online studies conducted synchronously (i.e., with a live experimenter present) have demonstrated feasibility, with minimal data loss, in assessing neurodevelopmental risk (Kelleher et al., 2020), looking time and learning (Smith-Flores et al., 2021) and cognition and memory (Sheskin and Keil, 2018) among infants and children. Importantly, both asynchronous and synchronous online studies of varied cognitive domains largely yield findings that replicate those from laboratory studies (e.g., Scott and Schulz, 2017; Sheskin and Keil, 2018; Rhodes et al., 2020; Smith-Flores et al., 2021).

Although this growing literature suggests the promise of online platforms for assessing cognitive development in the context of highly structured tasks, the validity on such methods for assessing dimensions of infant social and emotional functioning, such as parent-infant interaction, infant response to stress, and infant expressive and receptive communication skills, remains unknown. Whereas certain advantages (e.g., greater flexibility, more diverse participant pool) and disadvantages (e.g., poor internet connectivity, decreased experimental control, increased potential for distractions, see Su and Ceci, 2021) regarding virtual visits will be common across studies of cognitive and socioemotional development, some issues are unique. On the one hand, virtual visits may be particularly conducive to capturing infant and maternal social and emotional behaviors that are more ecologically valid because assessments take place in the familiar home environment without experimenters physically present. On the other hand, and precisely because the home environment is highly familiar, the effectiveness of virtual visits in eliciting infant response to stress may be reduced. Additionally, intensive assessments of mother-infant interaction using microanalytic coding schemes typically require video recording procedures that involve multiple cameras and pan/zoom/tilt functionality. Although two recent studies indicate feasibility of administering mother-infant interaction tasks *via* a virtual visit protocol (Gustafsson et al., 2021; Shin et al., 2021), feasibility was assessed subjectively (e.g., research assistant ratings), and validity was not assessed. With these issues in mind, we examined objective indicators of feasibility and validity of synchronous virtual visit procedures designed to assess mother-infant interaction, infant stress regulation, and infant language development.

With respect to infant socioemotional functioning, the Still-Face Paradigm (SFP; Tronick et al., 1978) is an established procedure to assess mother-infant interaction and infant responses to stress. The SFP involves three episodes, each typically 2 mins in length: (a) a “play” episode, in which the mother and infant interact without toys, (b) a “still face” episode, in which the mother looks at her infant but maintains a neutral facial expression and ceases interaction (i.e., vocalizations, touch), and (c) a “reunion” episode, in which the mother resumes interaction with her infant. The still face episode, which violates infants' expectations for reciprocal interaction, typically elicits a distress response. Use of microanalytic coding procedures, in which infant and maternal behaviors are coded continuously, permits a window into maternal and infant behavioral coordination during the play and reunion episodes (e.g., Sravish et al., 2013; Pratt et al., 2015; Busuito and Moore, 2017). Further, a meta-analysis of 39 studies indicated robust and expected effects of the SFP on infant behavior coded in a microanalytic manner: infant gaze to mother and positive affect decreased and infant negative affect increased from the play to still face episode, whereas infant positive affect and gaze to mother increased from still face to reunion (Mesman et al., 2009).

Importantly, Mesman et al. (2009) reported that SFP effects on infant behavior and affect were robust to procedural differences (e.g., length of episodes, use of transition interval in which mother turned away from infants between episodes) across studies, although consideration of the setting (i.e., lab vs. home) in which the procedure was carried out was not considered in this meta-analytic review. Whereas most studies using the SFP have been conducted in a controlled laboratory environment, Moore et al. (2001) conducted the SFP during home visits at 2, 4, and 6 months and reported expected changes in infant behavior (i.e., increases in negative affect and decreases in positive affect during the still face episode), thus supporting the use of the SFP in the home environment. Moreover, Gustafsson et al. (2021) reported that among 348 mother-infant dyads participating in a virtual visit procedure, including the SFP, 94–99% of videos passed data quality checks based on research assistant ratings. Although promising, objective evidence of feasibility (e.g., percentage of missing data) and validity of virtual SFP assessments is lacking.

In contrast to the lack of prior validation studies for remote assessments of mother-infant interaction during the SFP, several prior studies have reported feasibility and validity of assessing child language abilities using an online video conferencing format. Findings indicate that speech and language characteristics (e.g., mean length utterance, number of different words) among toddlers during play with a parent (Manning et al., 2020) and performance on a standardized language assessment among school-age children with language impairment (Sutherland et al., 2017) showed good feasibility, and reliability and/or validity of assessments did not differ significantly from data collected during face-to-face sessions. Ashworth et al. (2021), however, reported significantly higher verbal performance (assessed *via* the British Picture Vocabulary Scale, Third Edition [BPVS-3]) during online virtual visits vs. laboratory visits among school-aged children with Williams syndrome. Although these past studies indicate utility of conducting language assessments among toddlers and school-aged children *via* a virtual visit platform, we are unaware of prior work that has compared infant language assessments conducted *via* a synchronous virtual visit vs. an in-person laboratory format.

Complementing direct assessments of infant socioemotional and language functioning, assessing parents' perspectives about their virtual visit experiences is also needed. To date, the pros and cons of virtual visits for developmental research have been primarily discussed from the perspective of the researcher (see Su and Ceci, 2021). In this vein and in our own experience, advantages of virtual visits include greater re/scheduling flexibility, greater efficiency of cost and time, and better ability to recruit more (geographically) diverse samples, whereas disadvantages include diminished researcher control, greater dependence on parents to implement task procedures, and technical challenges that arise due to poor internet connectivity and/or shortcomings of participants' devices. Equally important, however, are parents' views about participation in developmental research using online platforms. Although there has been long-standing interest in clinical research (e.g., Yessis et al., 2012) and health care settings (e.g., Cleary and Edgman-Levitan, 1997) for assessing participants' or patients' perspectives of their experiences, such assessments are less common in developmental research (but see Kelleher et al., 2020; Maitre et al., 2021). Given the relative novelty of remote assessment methods in developmental research, and particularly the lack of such methods used to assess infant socioemotional and language development, mothers' perceptions of and preferences for visits of this type may provide a window into how and when such visits may be best used, as well as input for refining visit procedures in ways that not only increase data quality but also optimize participants' experience.

In the current study, we addressed three main objectives. First, we assessed whether infant and maternal SFP behaviors differed between laboratory and virtual visits on several objective metrics, including the frequency of missing data, distributions of behavioral codes, and expected changes in infant behavior across episodes. Second, we assessed whether infant receptive and expressive language differed between laboratory and virtual visits on similar metrics, including frequency of missing data and infant subtest scores. Third, we assessed mothers' perceptions of the virtual visit format and their preferences for virtual visits vs. in-person laboratory visits. We also conducted supplementary analyses to examine whether (a) the behavioral variables assessed during virtual visits and (b) maternal perceptions and preferences for virtual visits varied as a function of the dyad's prior experience with virtual visits.

## Method

### Participants

One hundred and nineteen infants (57 girls; 48%) and their mothers participated in a short-term longitudinal study from 3 to 12 months of age, in which the overarching goal was to investigate mother-infant interaction dynamics and attachment formation as predictors of infant physiological regulation and brain development. Families were recruited from local pediatric clinics, community organizations, and online forums serving families from a wide range of socioeconomic and racial/ethnic backgrounds. Families were excluded from participating if their infant had any known cardiac abnormalities, was born preterm (<37 weeks gestation), had birth complications and/or admission to the NICU, or had an MRI contraindication. Mothers were 13% Asian, 7% Black or African American, 73% White non-Hispanic, 4% Hispanic and 3% another race or more than one race. Forty-percent of mothers had completed a bachelor's degree, and 39% had received an advanced degree. The average annual family income was between $61,000 and $70,000.

Sample sizes and descriptive statistics for infant age and sex as a function of visit type at each time point are reported in Table 1. As shown in Table 1, 73 dyads participated in lab visits only, 13 dyads participated in virtual visits only, and 33 dyads participated in a combination of lab and virtual visits (labeled “hybrid” visit schedule). Chi-square analyses comparing visit type (lab vs. virtual visit at a given time point) by infant sex revealed one significant difference: At the 3-month time point, a higher proportion of female infants participated in virtual visits compared with laboratory visits, χ^2^ (1) = 4.20, *p* = 0.04. No other differences emerged for infant sex as a function of visit type. Because the one infant sex difference that emerged was based on small cell sizes (10 females vs. 3 males), and because no differences in the main study measures differed as a function of lab vs. virtual visits at 3 months (see Results), we did not consider infant sex further in our analyses. Additionally, *t*-tests for independent samples revealed no difference for infant age at each time point as a function of visit type, and one-way ANOVAs with visit schedule (lab only, virtual visit only, hybrid) as the between-subjects factor revealed no significant differences in maternal education and family income.

**Table 1:** Sample sizes and infant characteristics for mother-infant dyads participating in lab visits only, virtual visits only, and hybrid visits (lab and virtual).

**Visit schedule**	**3 months**	**6 months**	**9 months**	**12 months**
Lab visits only (*n* = 73)	49	67	62	58
Infant sex (% female)	59%	52%	52%	50%
Infant mean age (*SD*)	3.22 (0.29)	6.22 (0.38)	9.33 (0.43)	12.68 (0.48)
Virtual visits only (*n* = 13)	13	12	11	12
Infant sex (% female)	77%	75%	82%	75%
Infant mean age (*SD*)	3.34 (0.41)	6.28 (0.28)	9.26 (0.30)	12.38 (0.23)
Hybrid visits (*n* = 33)	33 (0)	26 (7)	13 (19)	0 (32)
Infant sex (% female)	27%	27%	25%	28%
Infant mean age (*SD*)	3.33 (0.35)	6.22 (0.25)	9.34 (0.35)	12.89 (0.91)
Total visits (*N* = 119)	95	112	105	102

*For hybrid visits, the number of dyads participating in lab visits at a given time point is shown first, followed by the number of dyads participating in virtual visits shown in parentheses*.

### Overview of Study Procedures

#### Laboratory Visits

Prior to COVID-19, infants and mothers participated in a 60-mins laboratory visit at 3, 6, and 9 months and a 90-mins visit at 12 months; infant brain scans (via magnetic resonance imaging [MRI]) during natural sleep were also conducted at 3 and 12 months. The laboratory visits included assessments of behavior and physiology (using 3-lead ECG wireless monitors) during a baseline session, play session, challenging puzzle task (12 months only), SFP (3, 6, 9 months only; Tronick et al., 1978), Strange Situation Procedure (12 months only; Ainsworth et al., 1978), as well as administration of Bayley cognitive and language subtests. Behavioral data from the SFP and Bayley language subtests were examined in this report. For the SFP, the infant was seated in an age-appropriate seat (e.g., bouncy seat, high chair). Mothers were provided with both verbal and written instructions about the SFP episodes, and a gentle knock on the door to the laboratory playroom signaled when to transition to the next episode. Following the mother-infant interaction sessions, a trained research assistant administered the Bayley-III Screening Test. Two professional cameras were mounted in opposite corners of the playroom; cameras had pan/tilt/zoom capabilities and were controlled and viewed from an observational booth adjacent to the playroom. All tasks were recorded for later review or scoring. Parents also completed a series of online questionnaires at each time point *via* Qualtrics.

#### Virtual Visits

During a 40-min virtual visit, we conducted assessments paralleling our laboratory assessments of (a) infant baseline physiology, (b) mother-infant play, (c) SFP (3, 6, 9 months only), (d) challenging puzzle task (12 months only), and (e) Bayley language subtests. Prior to the virtual visit, mothers were emailed a Zoom link as well as a list of materials needed during the visit (e.g., bouncy seat or high chair for SFP) and were reminded to charge the device (e.g., laptop, tablet, phone) they planned to use for the visit (see Garrisi et al., 2020; Smith-Flores et al., 2021, regarding recommendations for Zoom as platform for synchronous virtual visits). Zoom links were sent with the passcode function to protect participant privacy. Host and participant videos were switched to “on” and the waiting room feature was enabled. The visit coordinator recorded the session directly to the local computer (vs. Zoom cloud option) and data were subsequently uploaded to secure servers. The visit coordinator used the share screen function in Zoom to present slides that detailed instructions for each activity. After giving an overview of the visit activities and informing mothers of their right to request that the session or recording be stopped at any time (as was also done at the beginning of the laboratory visits), the visit coordinator started video recording and pinned the participant's video. During all activities, the visit coordinator turned off her video camera and microphone (except during the Baseline video- unmuted) and asked mothers to minimize their Zoom window so that infants would not be distracted by the screen. The share screen function was also used to share (a) the sea animals video for the baseline physiological assessment and (b) picture items for the Bayley receptive communication subtest. The visit coordinator worked with the mother to obtain the optimal video angle for each activity (e.g., capturing faces of both the mother and infant).

Participants were video recorded in a variety of rooms including living rooms, dining rooms, parent and infant bedrooms, and kitchens. Nonetheless, virtual visits maintained consistency with the lab visits in that an infant bouncy seat or high chair was used for the SFP, with mothers seated on the floor or in a chair accordingly. To provide an optimal camera angle for behavioral coding of the SFP, mothers and infants sat facing each other and slightly angled themselves toward the camera so that their faces were visible. To proactively minimize distractions, mothers were asked to silence their phones, turn off any electronic toys used during the play sessions, and limit the presence of pets or other family members as much as possible. Following the virtual visit, parents completed online questionnaires, which included a brief survey about maternal perceptions of the virtual visit.

### Measures

#### Behavioral Coding of the Still Face Paradigm (SFP)

The SFP was micro-coded for infant and maternal facial expressions, vocalizations, and directions of gaze using Datavyu 1.4.1, which allows for onset/offset coding of behaviors in real time and segmenting continuous codes by frame (33ms per frame). The current codes were adapted from existing coding systems on mother-infant interactions (Tronick et al., 1980; Braungart-Rieker et al., 1998; Moore et al., 2001; Moore and Calkins, 2004). For each code, categories were mutually exclusive and exhaustive. Data were coded as missing when the behavior of interest was not visible or audible, or when there was an interruption. With the following exception, the coding system and procedures were identical across laboratory and virtual visits. For the virtual visits only, we included “gaze at screen” as another category in the gaze code (see below) for both mothers and infants to capture degree to which the device used for the virtual visit was a distraction.

##### Infant Facial Expression

Codes were cry, frown, unalert (e.g., sleepy, yawn), alert neutral (e.g., wary, sober, bright, coo face), mild positive (e.g., simple or subtle smile), strong positive (e.g., broad smile, appearance of laughter), and other (e.g., sneeze, cough). *Infant vocalization* codes were cry, fuss, positive neutral (e.g., babble, coos), laughter, yawn, other (e.g., sneeze, cough, burp), and none. *Infant direction of gaze* codes were gazing at mother's face, gazing at mother's actions (e.g., following mother's moving fingers), gazing at other objects (e.g., gazing at mother's static legs or high chair), gazing away, eyes closed, and gazing at screen (virtual visits only).

##### Mother Facial Expression

Codes were angry, distressed, flat, interested, simple smile, broad smile, and other (e.g., yawn, sneeze, cough, sniff). *Mother vocalization* codes were infant-direct speech (i.e., baby talk with much change of modulation, bigger jump in pitch, or elongated vowels), adult speech (i.e., speaking normally as if she is talking to an adult with “regular” rhythm and intonation), playful noise (e.g., “raspberries” kissing sounds, animal sounds, “bounce-bounce-bounce”), rhythmic sounds or singing, laughter, demanding speech, whisper, other (e.g., yawn, sneeze, cough, grunt, sigh), and none. *Mother direction of gaze* codes were gazing toward infant's face, gazing at infant's body or interaction-related objects, gazing away, and gazing at screen (virtual visits only).

Two separate teams coded infant and maternal behaviors, and coders who participated in coding the laboratory visits also coded the virtual visits. Coders went through intensive training for one to three months and gained high reliability (*kappa* ≥ 0.80) on each code before proceeding with coding individual tapes. Within each coding team, different coders assessed behavior during the SFP across the three visits (3, 6, 9 months) whenever possible. Interobserver reliability, computed for 19 −25% of tapes that spanned across the entire coding process, were consistently satisfactory (kappa > 0.60; see McHugh, 2012) across all codes and time points (see Table 2). Of note, reliability was calculated using by-frame output where each unit represent 33 ms. A time buffer of 10 frames (~1/3 sec) was used to adjust for slight differences in coders' reaction times, and frames coded as missing by one or both coders were excluded from reliability calculations.

**Table 2:** Interobserver reliability statistics (Cohen's kappa) for infant and maternal behaviors in the Still Face Paradigm separately by visit type.

	**3 months**		**6 months**		**9 months**	
**Behavioral codes**	**Lab**	**Virtual**	**Lab**	**Virtual**	**Lab**	**Virtual**
**Infant behaviors**						
*N* (%) double-coded	17 (22%)	3 (23%)	18 (20%)	4 (22%)	15 (21%)	5 (19%)
Facial expression	0.74	0.76	0.69	0.72	0.72	0.77
Vocalization	0.79	0.67	0.83	0.76	0.83	0.70
Direction of gaze	0.76	0.70	0.82	0.74	0.78	0.73
**Maternal behaviors**						
*N* (%) double-coded	18 (23%)	3 (23%)	19 (21%)	4 (22%)	18 (25%)	5 (19%)
Facial expression	0.77	0.85	0.77	0.81	0.75	0.86
Vocalization	0.85	0.87	0.86	0.89	0.82	0.86
Direction of gaze	0.71	0.86	0.78	0.74	0.72	0.86

#### Infant Language Development

To assess infant receptive and expressive communication, we used the Bayley Scales of Infant and Toddler Development, Third Edition, Screening Test (Bayley, 2006a). The Bayley-III Screening Test is made up of items from the Bayley Scales of Infant and Toddler Development, Third Edition (Bayley, 2006b) and is a well-established and validated screening instrument designed to assess cognitive, language, and motor functioning of infants and young children (Bayley, 2006a). This screening instrument was normed on a US representative sample of 1,675 children aged 1–42 months and demonstrates excellent test-retest reliability, adequate internal consistency among subscale items, and good construct validity (Bayley, 2006a,b).

At both the virtual and laboratory visits, subtests assessing receptive communication (24 items) and expressive communication (24 items) were administered by a trained research assistant. The cognitive subtest was administered during the laboratory visits as well, but it was not deemed feasible for the virtual visits due to the required testing manipulatives, number of items, and complexity of instructing mothers to effectively administer the items. For infants in the age range participating in this study (3–12 months), items assessing receptive communication captured auditory acuity (e.g., responding to voices, discriminating sounds, localizing sounds), vocabulary development and comprehension (e.g., identifying objects or pictures that are referenced), and social referencing. Items assessing expressive communication captured preverbal communication (e.g., babbling, gesturing, joint referencing, turn taking) and vocabulary development (e.g., imitating words, naming pictures).

Following the Bayley (2006a) procedures, the infant's age determined the items to be administered, and administration ended when the infant failed four consecutive items. During the laboratory administration, the mother was present in the room. Administration for the language subtests took ~10–15 mins, although we note that the majority of the expressive communication items did not require formal administration and could instead be scored through “incidental observation” (i.e., observing the infant during any part of the laboratory or virtual visit for expressive behaviors that satisfied the scoring criteria for a given item).

Several adaptations in administration procedures were made for the virtual visits. Prior to the visit, the visit coordinator informed the mother about materials needed for testing; these materials were typical items that a family with an infant would have at home (e.g., blocks, ball, spoon). To administer the Bayley-III Screening language subtests virtually, the visit coordinator (who had received extensive training in administration and scoring procedures) would observe the infant's verbal and communicative behavior throughout the virtual visit to score items relevant to expressive communication and would guide the mother through the standard administration of items that could not be scored through incidental observation. In doing so, the visit coordinator provided verbal and written (through PowerPoint slides displayed *via* shared screen) instructions. For items in which a stimulus book was needed, we created comparable testing stimuli using open-source images available online, and the visit coordinator would show the pictures in PowerPoint slides *via* the share screen function in Zoom. The mother would verbally prompt the infant (e.g., *Show me the bird*.) The mother was instructed to indicate to the visit coordinator whether the infant was pointing or clearly looking at the item she mentioned. To minimize distractions, the visit coordinator also instructed mothers to minimize the Zoom screen during items where it was not needed.

For both laboratory and virtual visits, the researcher scored the receptive communication items as they were administered and reviewed the video recording following a given visit for any items for which questions arose. Scoring of expressive communication items varied slightly during laboratory vs. virtual visits. During the laboratory visits, the researcher administering the Bayley observed the infant *via* a video monitor during the different sessions (e.g., baseline, play, SFP), made detailed notes and scored items on the Bayley-III scoring form while carrying out these “live” observations of the infant; video recordings were reviewed following the visit if questions arose about scoring specific items. During the virtual visits, because one researcher managed all roles (e.g., providing mothers with task instructions, managing the video recording, administering Bayley items), the expressive items were scored almost exclusively by reviewing the video recording following the visit; paralleling scoring procedures from the laboratory visits, detailed notes were also entered on the scoring form. Further, a doctoral-level researcher with seven years of Bayley-III experience reviewed all Bayley procedures for all laboratory and virtual visits and corrected scores as needed to ensure accuracy of administration and scoring.

#### Maternal Perceptions of Virtual Visits

Following research visits at each time point, mothers completed a series of questionnaires online. Shortly after the onset of the virtual visits, we added items about maternal perceptions of the virtual visits to the questionnaire protocol, which mothers also completed at each time point in which they participated in a virtual visit. Mothers rated the following two items on a five-point scale: (a) Was the virtual visit easy for you to manage? (1 = *very easy* to 5 = *very challenging*), and (b) How well do you think the virtual visits vs. in-person visits capture you and your baby's typical interactions? (1 = *much more typical during the virtual visit* to 5 = *much more typical during the in-person visit*; N/A option available for families who had not experienced an in-person visit). Mothers were also asked whether they would choose a virtual or in-person visit if they had the choice (forced choice: *virtual, in person, no preference*). If a preference for virtual or in-person visits was indicated, the mother was asked to describe her reason/s for the given preference.

### Data Analytic Plan

Below we outline the analyses conducted to address the three main aims of this report. In addition, supplementary analyses are presented after the main analyses and assessed whether infant and maternal data collected during virtual visits at 6, 9, and 12 months varied as function of infant- mother dyads' prior experience with virtual visits within the context of this study.

#### Infant and Maternal Behavior (SFP)

Our first research objective was to assess the feasibility (i.e., rates of missing data) and validity (i.e., proportional breakdown of categories within a given code, as well as change in key infant behaviors) of the virtual visits vs. lab visits in capturing infant and maternal behavior during the SFP. We assessed two types of missing data for mother-infant behavioral data from the SFP. First, at each time point, we compared proportions of data from laboratory vs. virtual visits that were completely missing (i.e., the observational assessment was attempted or fully conducted but could not be coded) using *z* tests. Second, for SFP sessions that were deemed codeable, we considered the proportion of missing data for a given code when parts of the recording were not codeable due to the camera angle (e.g., infant or mother's face out of camera view) or interruptions (e.g., a third person entered the room; a noise distracted baby or mother). Because the proportion scores showed high levels of skewness and kurtosis, we used the Mann Whitney *U*-test for independent samples to compare the distributions of missingness for lab and virtual visits; this nonparametric test, which does not assume normal distribution of the dependent variable, was more appropriate than an independent samples *t*-test.

Next, to test whether infant and maternal behaviors varied by visit type, we first computed proportion scores for maternal and infant behaviors as the number of frames for a given behavior (e.g., gazing at mother's face) divided by the total number of frames coded for that particular behavioral category (e.g., infant direction of gaze), excluding frames that were coded as missing. We then tested whether proportion scores were different across lab and virtual visits using Mann-Whitney *U*-test s for independent samples. Given the multiple comparisons made (3 episodes × 3 time points), we applied a Bonferroni correction of *p* < 0.006 (0.05 divided by 9) for each infant and maternal code (facial expression, vocalization, direction of gaze).

Lastly, to assess whether expected patterns of change in infant negative affect, positive affect, and gaze varied as a function of visit type (and in accordance with prior work, see Mesman et al., 2009), we computed the following composite scores within episode and time point: *infant negative affect* (i.e., mean of infant negative facial affect [cry + frown] and vocalization [cry + fuss] proportion scores), *infant positive affect* (i.e., sum of infant mild positive and strong positive facial affect proportion scores), and *infant gaze toward mother* (i.e., sum of infant gaze at mother's face and gaze at mother's actions). To be comparable to lab visits, we excluded the “gaze to screen” code assessed during virtual visits when computing proportion scores for infant gaze. Repeated measures ANOVA were conducted separately for each dependent variable with SFP episode as the repeated/within-subjects factor and visit type as the between-subjects factor. Because infants' participation in lab vs. virtual visits changed across time, it was not possible to include time point as a second repeated measure and, thus, separate models were tested at 3, 6, and 9 months.

#### Infant Receptive and Expressive Communication (Bayley-III Screening Test)

Our second research objective was to assess the feasibility (i.e., rates of missing data) and validity (i.e., infant performance) of the virtual visits vs. lab visits in capturing infant receptive and expressive communication. For each of the language subtests and at each time point, we compared proportions of data from lab vs. virtual visits that were completely missing (i.e., the Bayley subtest was attempted or fully administered but could not be scored due to missing items) using *z* tests. Second, to compare scores from lab and virtual visits, we conducted *t*-tests for independent samples by subtest and time point. For both sets of tests, we used a Bonferroni correction of *p* < 0.0125 (0.05 divided by 4 time points) to adjust for multiple comparisons across time.

#### Maternal Perceptions and Preferences

Our third objective was to assess maternal perceptions of the virtual visit format and preferences for virtual vs. in-person, lab visits. Across all time points, 104 survey responses were obtained from 44 mothers. Using all maternal report data available, we conducted single sample *t*-tests (for maternal “ease” and “typical” ratings) at each time point to determine whether mean ratings significantly differed from the midpoint of the scale (i.e., value of 3 = “neutral” or “about the same”). With respect to maternal visit preferences (prefer virtual visit, prefer lab visit, no preference), we conducted a one-sample proportion test at each time point to assess whether the proportion of mothers who reported preference for virtual and lab visits, respectively, differed significantly from 0.33 (the proportion expected by chance).

## Results

### Infant and Maternal Behavior During the Still Face Paradigm

#### Missing Data

We assessed two types of missing data. The proportions of cases completely missing SFP data at each time point are shown in Table 3. At each time point, a chi-square analysis compared rates of missing data by visit type (lab vs. virtual). Although all comparisons were nonsignificant, reasons for missingness varied by visit type. As shown in Table 3, infant distress was a frequent reason for missingness during lab visits, in particular. With respect to SFP data that were coded, we also considered proportion of missing data for a given code at a given time point due to sections of the session that could not be coded (e.g., poor camera angle, mother blocked infant from view). As shown in Table 4, data were missing at higher proportions during virtual vs. lab visits, with 10 of 18 tests indicating a significant difference by visit type. Differences in proportions of missingness emerged for infant facial expression at all three time points and for mother facial expressions at two of the three time points. Facial expression missingness was also greatest in absolute terms at all time points and for both infants and mothers.

**Table 3:** Dyads with data missing for the Still Face Paradigm as a function of visit type.

**Reason for missingness**	**Total *N***	**3 months**	**6 months**	**9 months**
Lab visits	19 (7.6%)	10 (12%)	4 (4%)	5 (7%)
Equipment failure	4	2	1	1
Infant distress	12	7	3	4
Time constraints	1	1	0	0
Virtual visits	4 (6.5%)	0 (0%)	1 (5%)	3 (10%)
Experimenter error	1	0	1	0
Infant distress	1	0	0	1
Screen distraction	2	0	0	2

**Table 4:** Proportion of missing data during the Still Face Paradigm by behavioral code, time point and visit type.

**Time point**	**Lab visits**	**Virtual visits**		
**Behavioral code**	***M* (*SD)***	***M* (*SD)***	***z* statistic**	***p* value**
**3 months**				
Infant facial expression	0.004 (0.018)	0.103 (0.163)	4.66	<0.001
Infant vocalization	0.001 (0.004)	0.006 (0.016)	1.13	0.259
Infant gaze	0.004 (0.017)	0.066 (0.113)	3.92	<0.001
Mother facial expression	0.030 (0.051)	0.123 (0.117)	3.36	<0.001
Mother vocalization	0.001 (0.004)	0.013 (0.022)	3.19	0.001
Mother gaze	0.012 (0.029)	0.029 (0.040)	1.27	0.205
**6 months**				
Infant facial expression	0.017 (0.040)	0.093 (0.117)	4.44	<0.001
Infant vocalization	0.009 (0.077)	0.001 (0.003)	−0.13	0.895
Infant gaze	0.009 (0.025)	0.025 (0.063)	2.40	0.016
Mother facial expression	0.059 (0.078)	0.131 (0.206)	1.70	0.089
Mother vocalization	0.010 (0.077)	0.005 (0.009)	1.49	0.135
Mother gaze	0.029 (0.065)	0.019 (0.031)	−0.54	0.593
**9 months**				
Infant facial expression	0.017 (0.038)	0.078 (0.064)	5.14	< .001
Infant vocalization	0.002 (0.005)	0.003 (0.008)	−0.80	0.423
Infant gaze	0.009 (0.013)	0.022 (0.030)	1.57	0.118
Mother facial expression	0.061 (0.066)	0.128 (0.091)	4.35	<0.001
Mother vocalization	0.001 (0.002)	0.006 (0.010)	2.74	0.006
Mother gaze	0.031 (0.048)	0.069 (0.111)	2.98	0.003

*Mann-Whitney U-tests were performed to test differences between lab visits and virtual visits in proportion scores for a given code at a given time point. Standardized test statistics and p values are shown in the table*.

#### Distributions of Behavioral Codes

**Figure 1:**
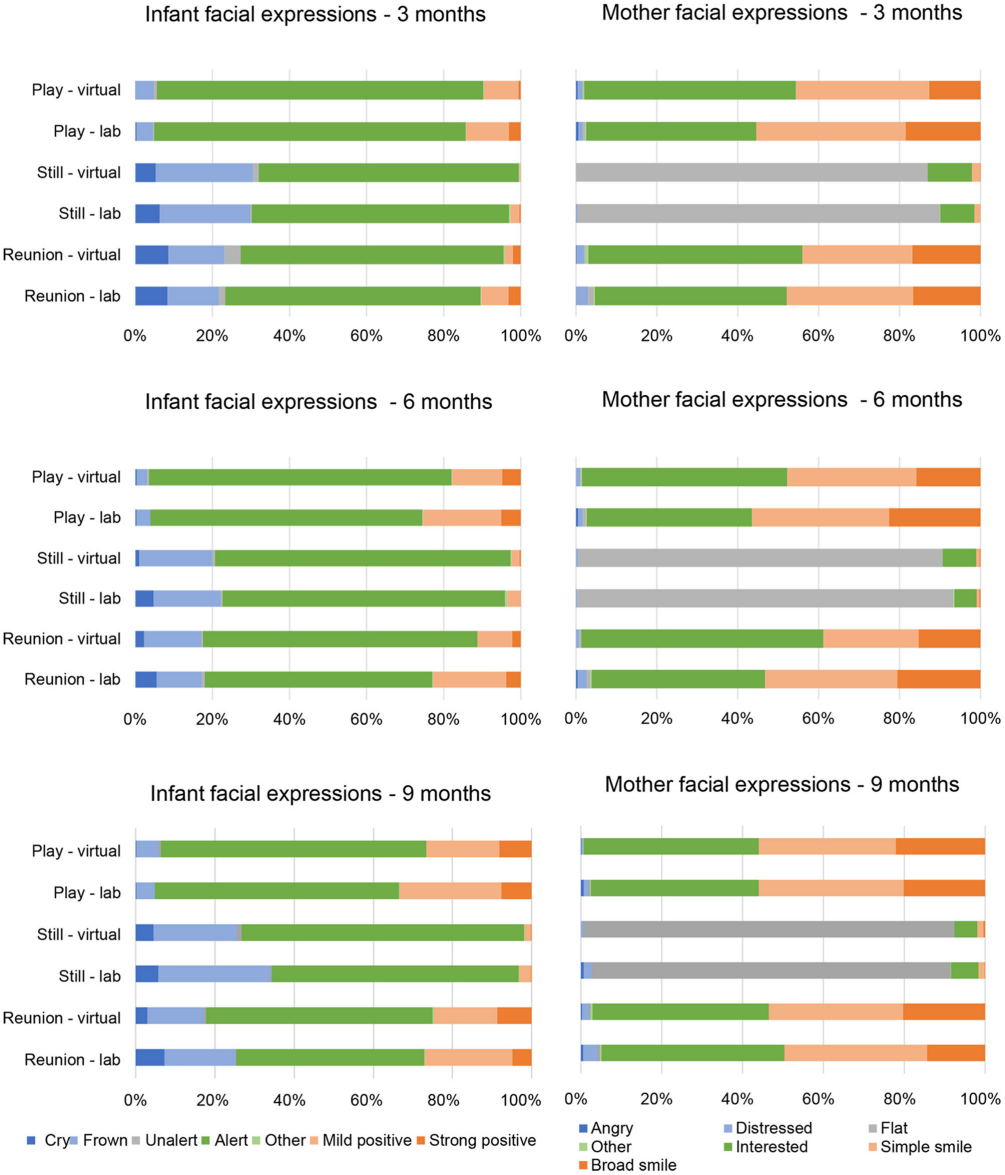
Infant and mother facial expressions by Still Face episode, visit type and time point.

**Figure 2:**
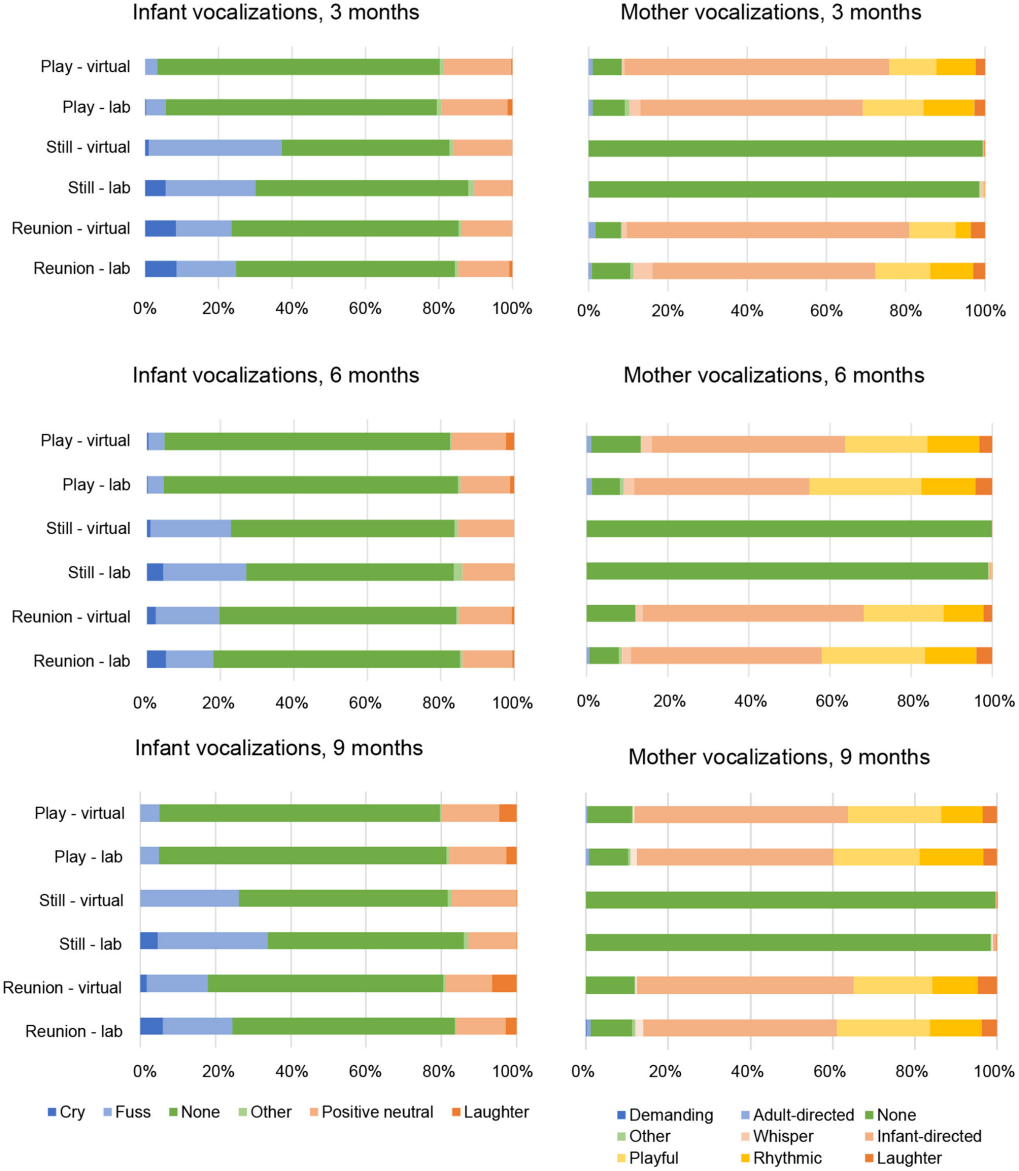
Infant and mother vocalizations by Still Face episode, visit type and time point.

**Figure 3:**
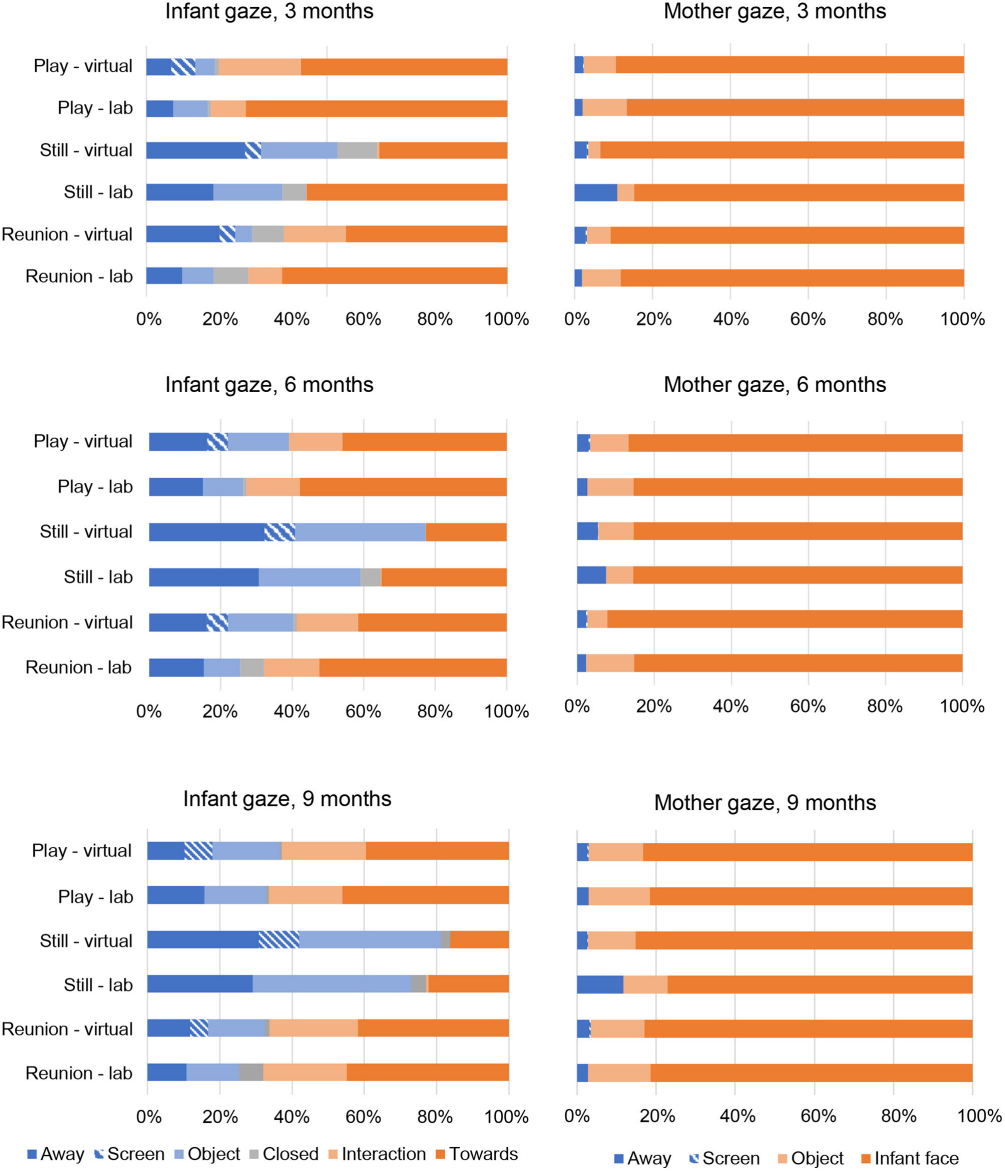
Infant and mother gaze by Still Face episode, visit type and time point.

Within each episode of the SFP and each time point, we plotted the proportion of maternal and infant behaviors for facial expression (see Figure 1), vocalization (see Figure 2) and gaze (see Figure 3) codes, respectively.

As shown in Figure 1, “alert” was the most frequent infant facial expression across time points and SFP episodes. Mothers showed high frequencies of “interested,” “simple smile,” and “broad smile” during the play and reunion SFP episodes and high frequencies of “flat” during the still episode at each time point. Of the 63 Mann-Whitney *U*-tests conducted for infant facial expressions (7 codes × 3 episodes × 3 time points), one comparison was significant at *p* < 0.006 (i.e., correction for multiple comparisons). At 6 months, infant showed more unalert facial expressions in the play episode (*z* = −3.89, *p* < 0.001) during virtual vs. lab visits. Of the 63 tests conducted for mother facial expressions (7 codes × 3 episodes × 3 time points), one was significant at *p* < 0.006. At 6 months, mothers showed more interested facial expressions (*z* = −2.82, *p* = 0.005) in the reunion episode during virtual vs. lab visits.

As shown in Figure 2, “none” was the most frequent infant vocalization code across time points and SFP episodes, whereas mothers showed relatively high frequencies of “infant-directed,” “playful,” and “rhythmic” vocalizations during the play and reunion episodes at each time point. Of the 54 Mann-Whitney *U*-tests conducted for infant vocalizations (6 codes × 3 episodes × 3 time points), one was significant at *p* < 0.006. Infants engaged in more crying during the still episode at 9 months (*z* = −3.04, *p* = 0.002) in lab vs. virtual visits. Of the 81 tests conducted for mother vocalizations (9 codes x 3 episodes x 3 time points), two were significant at *p* < 0.006. Mothers were more likely to be silent (i.e., vocalizations coded as “none”) during the still episode at 6 months in virtual vs. lab visits (*z* = −3.285, *p* = 0.001) and more likely to exhibit “other” vocalizations (e.g., yawn, sneeze, cough) during the reunion episode at 9 months in lab vs. virtual visits (*z* = −2.92, *p* = 0.003).

As shown in Figure 3, and at each time point, infants' gaze toward the mother's face or actions was predominant during both the play and reunion episodes, whereas infant gaze was more evenly divided among gazing away, gazing at object, and gazing at mother's face during the still episode. At each time point and across episodes, mothers showed high frequencies of gazing at infant's face. Note that “gaze at screen” was only coded during the virtual visits to capture infant or maternal distraction with the device used for the virtual visit. Of the 45 Mann-Whitney *U*-tests conducted for infant gaze (5 codes [excluding “gaze at screen”] × 3 episodes × 3 time points), and the 27 tests conducted for maternal gaze (3 codes [excluding “gaze at screen”] × 3 episodes × 3 time points), none were significant at *p* < 0.006.

#### Change in Infant Behavior Across SFP Episodes

**Figure 4:**
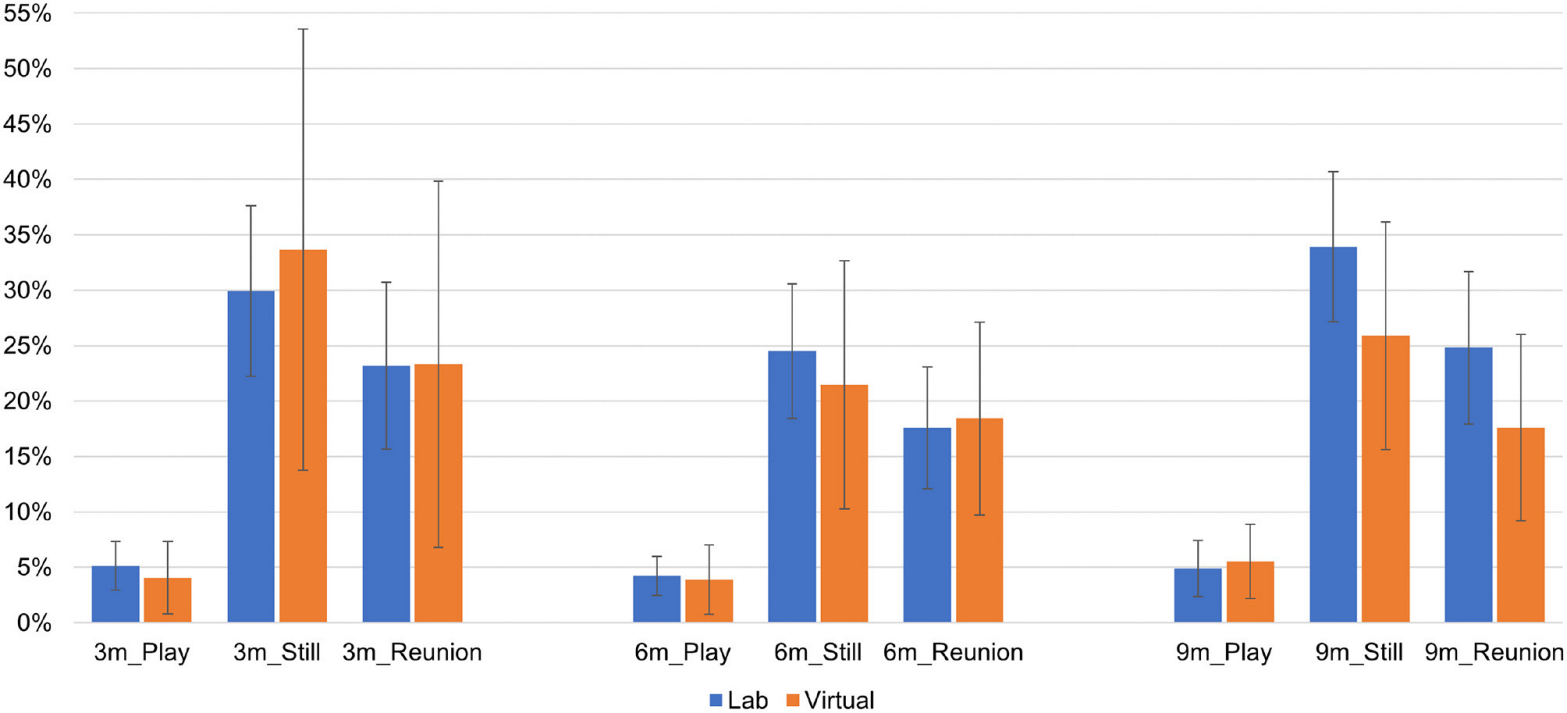
Infant negative affect at each time point as a function of Still Face episode and visit type.

**Figure 5:**
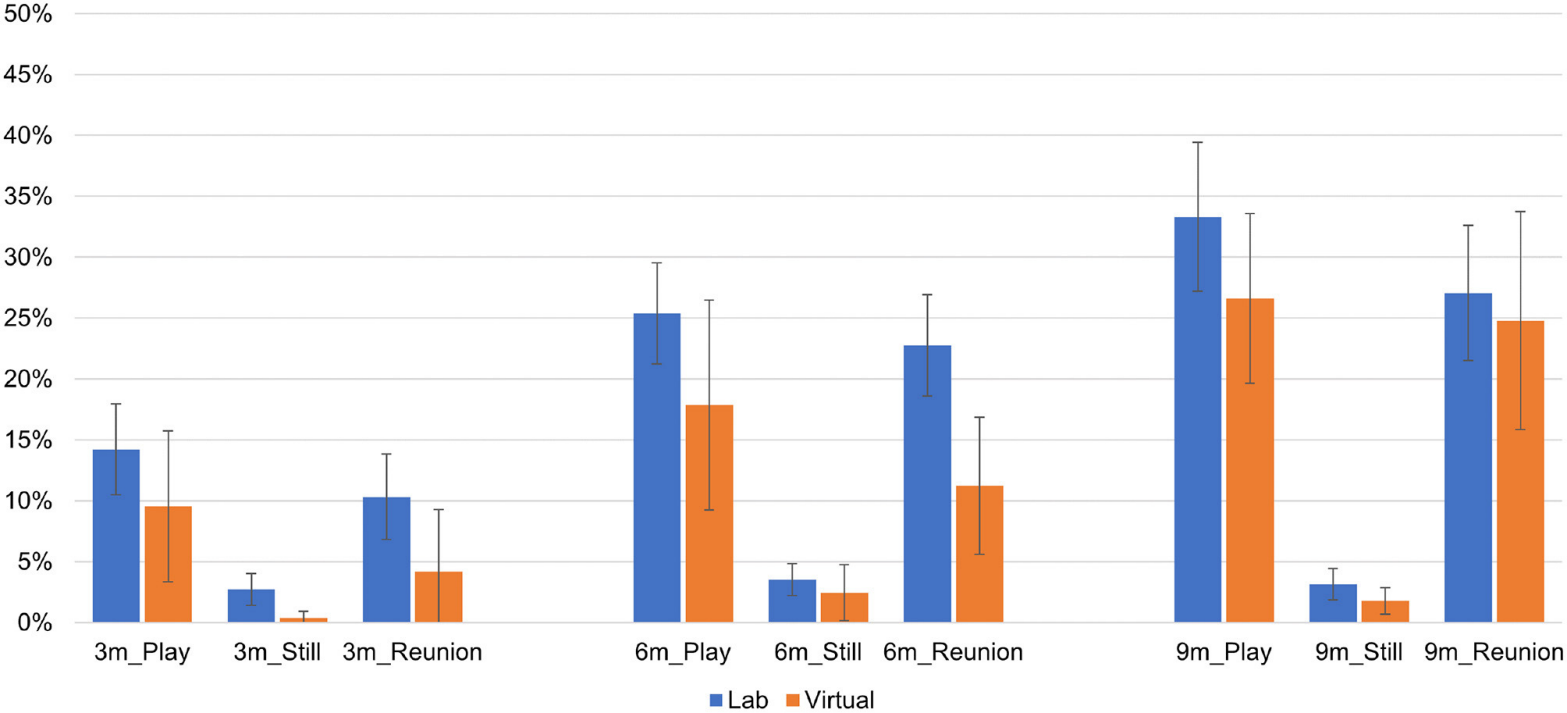
Infant positive affect at each time point as a function of Still Face episode and visit type.

**Figure 6:**
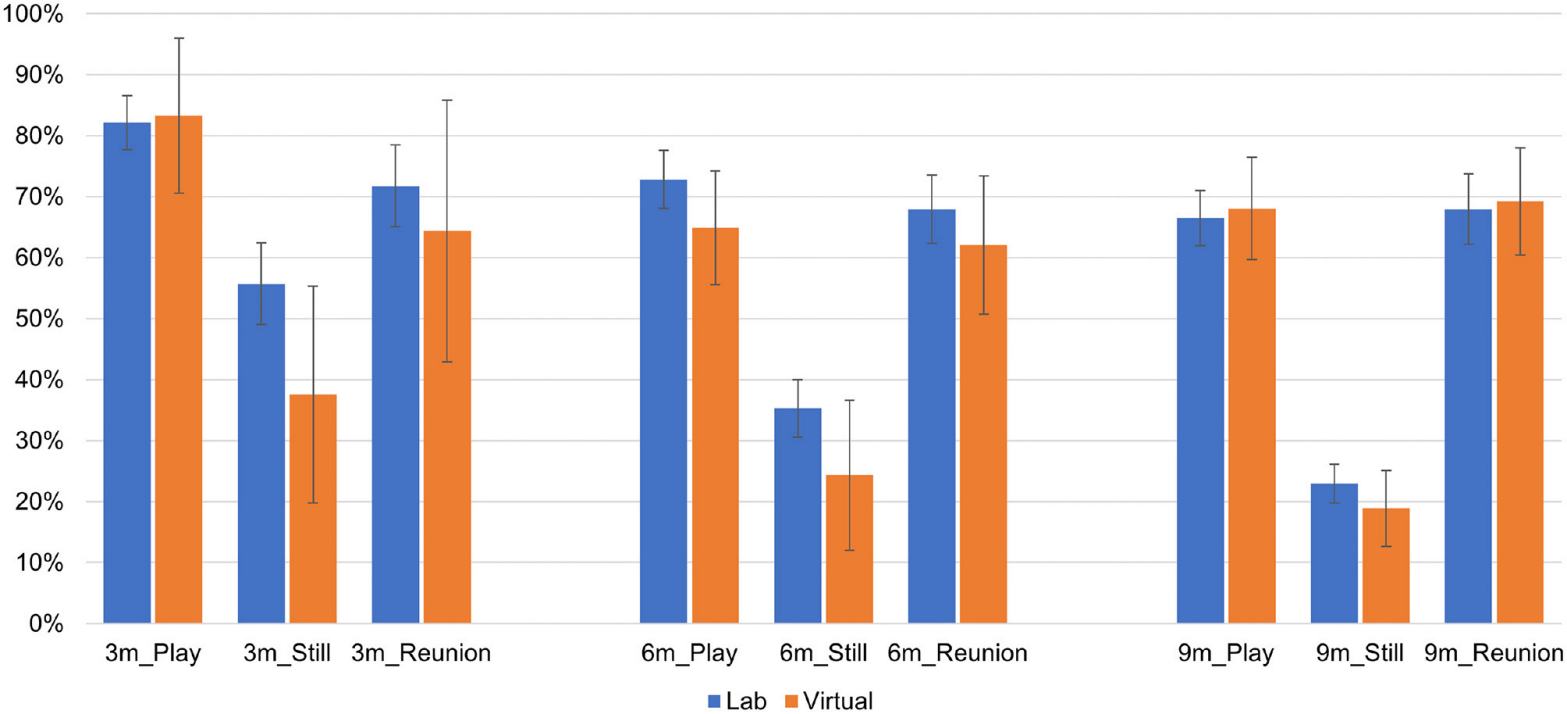
Infant gaze to mother at each time point as a function of Still Face episode and visit type.

Next, we report mean proportion scores and 95% confidence intervals for infant negative affect (see Figure 4), infant positive affect (see Figure 5), and infant gaze toward mother (see Figure 6) as a function of SFP episode, visit type and time point. As shown in Table 5, the main effect of episode was significant at *p* < 0.001 for each infant composite at each time point, and the main effect of episode did not vary as a function of visit type (i.e., Episode × visit type interaction was nonsignificant in all cases).

**Table 5:** Tests of infant behavioral change at each time point as a function of SFP episode and visit type.

						**Episode mean differences**		
**Dependent variables**	**Effects**	***MS***	***df***	***F***	***p***	**Still—Play**	**Still—Reunion**	**Reunion—Play**
**Negative affect**								
3 months	Episode	0.851	2	21.16	<0.001	0.27^***^	0.09	0.19^**^
	Visit type	0.003	1	0.02	0.884			
	Episode × visit type	0.007	2	0.17	0.845			
6 months	Episode	0.576	2	18.70	<0.001	0.19^***^	0.05	0.14^***^
	Visit type	0.003	1	0.038	0.847			
	Episode × visit type	0.006	2	0.19	0.824			
9 months	Episode	1.224	2	37.25	<0.001	0.25^***^	0.09^**^	0.16^***^
	Visit type	0.139	1	1.48	0.227			
	Episode × visit type	0.045	2	1.37	0.258			
**Positive affect**								
3 months	Episode	0.118	2	10.63	<0.001	−0.10^***^	−0.06^*^	−0.05
	Visit type	0.064	1	2.66	0.107			
	Episode × visit type	0.004	2	0.37	0.691			
6 months	Episode	0.564	2	32.60	<0.001	−0.19^***^	−0.14^***^	−0.05
	Visit type	0.202	1	4.916	0.029			
	Episode × visit type	0.042	2	2.41	0.093			
9 months	Episode	1.719	2	81.97	<0.001	−0.28^***^	−0.24^***^	−0.04
	Visit type	0.070	1	0.99	0.321			
	Episode × visit type	0.016	2	0.76	0.469			
**Gaze to mother**								
3 months	Episode	1.45	2	34.45	<0.001	−0.36^***^	−0.21^***^	−0.15^**^
	Visit type	0.219	1	1.79	0.185			
	Episode × visit type	0.103	2	2.44	0.090			
6 months	Episode	2.772	2	84.96	<0.001	−0.39^***^	−0.35^***^	−0.04
	Visit type	0.307	1	2.98	0.087			
	Episode × visit type	0.010	2	0.30	0.742			
9 months	Episode	5.750	2	272.52	<0.001	−0.46^***^	−0.48^***^	0.01
	Visit type	0.001	1	0.014	0.907			
	Episode × visit type	0.020	2	0.929	0.397			

* *p < 0.05*,

** *p < 0.01*,

*** *p < 0.001*.

*Post-hoc* analyses of the episode main effect revealed expected changes in infant behavior, such that (a) infant negative affect was significantly lower during the play episode vs. still and reunion episodes, with a difference between the still and reunion episodes (more negative affect in the still episode) emerging only at 9 months, (b) infant positive affect was significantly lower during the still episode vs. the play and reunion episodes, with no difference between these latter episodes, and (c) infant gaze toward mother was significantly lower during the still episode vs. play and reunion episodes, with a difference between play and reunion (less gaze toward mother during reunion) present only at 3 months. The main effect of visit type was significant in one instance. Across all episodes of the SFP, infants exhibited more positive affect during lab visits compared with virtual visits at the 6-month time point. All other main effects of visit type were nonsignificant.

### Infant Receptive and Expressive Communication

#### Missing Data

Within each time point, comparisons of proportions of infants missing data on receptive or expressive language subtests at lab vs. virtual visits indicated significantly higher proportions of missingness on receptive language subtests at virtual vs. lab visits at 3, 6, and 9 months (see Table 6). Proportions of missing data on (a) receptive language at 12 months and (b) expressive language at any time point did not differ significantly by visit type. Infant distress/fatigue was the most common reasons for missing scores on the Bayley-III language subtests during the lab visits, whereas difficulty administering and/or scoring items (specific to the receptive language subtest) was the most common reason for missing data during the virtual visits. Additional although much less common reasons for missing language data for both lab and virtual visits included equipment failure, parental time constraints, and experimenter error.

**Table 6:** Infant receptive and expressive communication: missing data and infant performance as a function of visit type and time point.

**Language assessment**	**Lab visits**	**Virtual visits**		
**Missing data**	***n*** **(%)**	***n*** **(%)**	***z*** **test**	***p*** **value**
Receptive language				
3 months	6 (7%)	4 (31%)	2.56	0.010
6 months	6 (7%)	6 (32%)	3.20	0.001
9 months	5 (7%)	8 (27%)	2.81	0.005
12 months	15 (26%)	17 (41%)	1.55	0.122
Expressive language				
3 months	2 (2%)	0 (0%)	−0.57	0.569
6 months	7 (8%)	1 (5%)	−0.36	0.719
9 months	3 (4%)	1 (3%)	−0.16	0.872
12 months	7 (12%)	2 (5%)	−1.26	0.208
**Infant performance**	***M*** **(*****SD*****)**	***M*** **(*****SD*****)**	***t*** **-test**	***p*** **value**
Receptive language				
3 months	3.13 (1.15)	2.78 (1.30)	−0.86	0.391
6 months	5.93 (1.45)	5.23 (0.93)	−1.69	0.094
9 months	7.23 (1.25)	7.09 (1.44)	−0.43	0.666
12 months	9.00 (1.86)	9.92 (2.77)	1.64	0.107
Expressive language				
3 months	4.16 (1.08)	4.46 (0.78)	0.95	0.343
6 months	5.24 (1.35)	5.44 (0.92)	0.63	0.533
9 months	7.21 (1.81)	7.86 (1.30)	2.03	0.047
12 months	10.69 (2.08)	10.63 (2.08)	−0.14	0.890

#### Receptive and Expressive Communication Scores

Mean and standard deviations for infant receptive and expressive communication scores are shown by time point and visit type in Table 6. Across all time points and for each subtest, *t*-tests for independent samples revealed no significant differences at *p* < 0.0125 (Bonferroni correction) in infant language scores as a function of virtual vs. lab visits (see Table 6).

### Maternal Perceptions and Preferences

**Figure 7:**
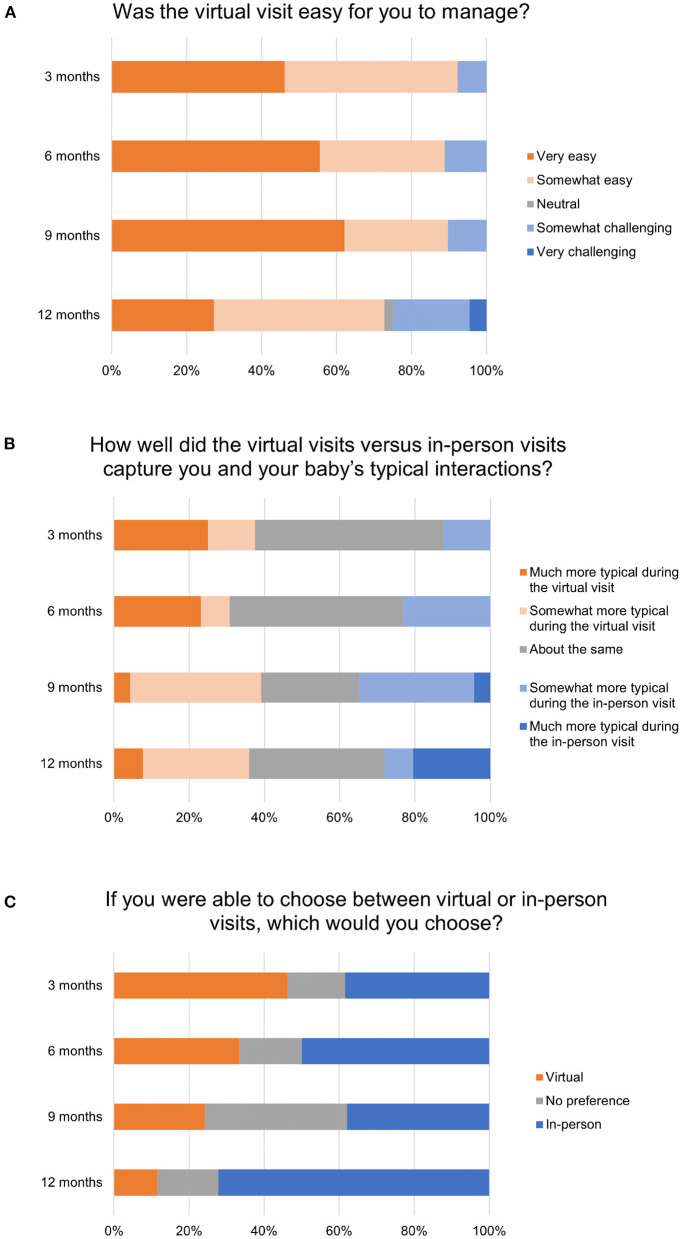
Distributions of mothers' **(A)** ratings of ease of virtual visits, **(B)** ratings of how well the virtual visits captured typical interactions between the mother and her infant, and **(C)** preferences for virtual versus in-person visits. Exact wording of questionnaire items are shown above. Maternal responses to each item are displayed by time point.

Mothers' virtual visit ratings and visit preferences as a function of time point and visit type are shown in Figure 7. As a preliminary step, we assessed whether demographic characteristics (infant sex, maternal education, family income) were related to maternal virtual visit perceptions or preferences. We used maternal reports following the mother's first virtual visit (regardless of time point, *n* = 44), and all associations were nonsignificant.

Next, to assess whether maternal perceptions of virtual visits were significantly different than a “neutral” rating, single-sample *t*-tests were conducted within each time point and indicated a significant difference for maternal ratings of visit ease at each time point (see Figure 7A). Mothers, on average, rated the virtual visits on the “easy” end of the scale compared with the scale mid-point (3 = “neutral”): *t*_(12)_ = −5.52, *p* < 0.001 (3 months), *t*_(17)_ = −5.83, *p* < 0.001 (6 months), *t*_(28)_ = −8.05, *p* < 0.001 (9 months), and *t*_(43)_ = −3.86, *p* < 0.001 (12 months). For maternal ratings of how well the lab vs. virtual visits captured typical interactions between the mother and her infant (see Figure 7B), maternal mean ratings were not significantly different from the scale mid-point (3 = “about the same”).

One-sample proportion tests of maternal preferences (prefer virtual visit, prefer lab visit, no preference, see Figure 7C) indicated a significant difference in maternal preferences at 12 months only, with 72% of mothers indicating preference for in-person visits, whereas 11% and 16% indicated preference for virtual visits or no preference, respectively, *z* = 5.40, *p* < 0.001. If a visit preference was indicated, we also asked mother to provide a brief explanation for her preference. Preferences for virtual visits included (a) *convenience* for mothers and families to schedule a visit without disruption to family routines (*n* = 10), (b) *safety* of families and their decreased potential exposure to COVID-19 (*n* = 6), and (c) *familiarity*, with a small group of mothers reporting their infant's behavior is more natural in the home environment vs. a new setting with strangers (*n* = 5). Preferences for in-person lab visits included (a) *decreased distractions* to mothers and infants (*n* = 25), (b) *greater confidence* in conducting visit procedures with the help of research staff, especially in handling technology (*n* = 11), and (c) *desire for face-to-face interaction* vs. interacting through a screen (*n* = 8). Mothers of older infants (12 months) were much more likely to report their preference for in-person lab visits due to decreased distractions and greater assistance by research staff in carrying out study procedures.

Given the difference that emerged in maternal preferences at 12 months, we conducted two sets of follow-up analyses. First, using mothers' reports completed after their first virtual visit only, we conducted one-way ANOVAs with time point as the between-subjects factor with maternal “ease” and “typical” ratings, respectively, as the dependent variable. These analyses allowed direct tests of whether maternal perceptions differed as a function of time point and also controlled for mothers' prior experience with virtual visits. For maternal ratings of the degree to which the virtual visit was easy vs. difficult, the main effect of time point was marginally significant, *F*(3) = 2.37, *p* = 0.085, although a planned contrast revealed that mothers reported less ease of virtual visits at 12 months (*M* = 2.43, *SD* = 1.28) compared with maternal reports at the other three time points combined (3 months: *M* = 1.69, *SD* = 0.85; 6 months: *M* = 1.33, *SD* = 0.52; 9 months: *M* = 1.64, *SD* = 0.92), *t*_(40)_ = 2.65, *p* = 0.011. No time-point effects emerged for maternal reports of how typical their interaction with their infant was during virtual vs. lab visits.

Second, we assessed whether maternal preferences for lab visits at the 12-month time point may have been due to COVID timing (e.g., the 12-month reports were collected later in the pandemic). To explore this possibility, we assessed whether there was a significant difference in COVID timing (computed as number of days between the date of the maternal report and March 13, 2020, marking the beginning of COVID-related shutdown) as a function of visit time point, and this test was nonsignificant, *F*(3) = 1.65, *p* = 0.182. At each time point, we also conducted a one-way ANOVA to examine whether COVID timing differed as a function of maternal visit preferences, and all tests were nonsignificant.

### Supplementary Analyses

Among the 19 dyads participating in a virtual visit at 6 months, 12 dyads had participated in a virtual visit at 3 months. Of the 30 dyads participating in a virtual visit at 9 months, 18 had participated in one or more prior virtual visits (11 dyads at 3 and 6 months; 7 dyads at 6 months). Of the 44 dyads participating in a virtual visit at 12 months, 29 had participated in one or more prior virtual visits (11 dyads at 3, 6, and 9 months; 7 dyads at 6 and 9 months; 12 dyads at 9 months). Given that participants differed in the degree to which they had previously experienced virtual visits, we conducted supplemental analyses that paralleled the main analyses reported above to assess whether key study variables (i.e., proportions of missing data, infant and maternal behaviors during the SFP, infant receptive and expressive language) differed as a function of prior virtual visit exposure (present vs. absent; 6-, 9-, and 12-month time points) or number of prior virtual visits (9- and 12-month time points). Correcting for multiple comparisons, no significant differences emerged as a function of prior virtual visit experience.

Maternal perceptions and preferences related to virtual visits were also examined as a function of prior exposure to virtual visits. We conducted a series of independent *t*-tests at each time point (6, 9 and 12 months, respectively) to assess whether maternal “ease” and “typical” ratings differed as a function of the mother's prior experience with virtual visits. At the 9- and 12-month time points, Kendall's tau-b (τ_b_) correlations were tested to assess whether the number of prior virtual visits was associated with maternal ratings. All tests were nonsignificant. Chi-square tests were conducted to assess differences in visit preference by prior virtual visit experience. At 9 months, mothers indicated greater preference for lab visits when they had not had prior exposure to virtual visits (total *n* = 11, 73% lab, 0% virtual, 27% no preference), whereas preferences were more evenly distributed among mothers with prior experience (total *n* = 18, 17% lab, 39% virtual, 44% no preference), χ^2^ (2) = 10.47, *p* = 0.005. Maternal visit preferences at 6 and 12 months did not significantly differ as a function of prior experience.

## Discussion

The COVID-19 pandemic has required researchers to adapt to a unique set of circumstances, and such adaptations are paving the way for innovative research methods that will likely continue to be used and developed. The current project adds to growing evidence for the feasibility and validity of virtual visits. Complementing existing online paradigms that have been used to assess infants' cognitive development (Scott et al., 2017; Tran et al., 2017; Smith-Flores et al., 2021), our study focused on the use of established assessments to capture infant socioemotional and language development during the first year of life. Further, by conducting assessments at multiple time points, we were able to explore whether virtual visits were more or less feasible or valid at different periods during the first year. Lastly, we gained insight into maternal perspectives on virtual visits and how such perspectives differed by infant age.

Our first objective was to assess the feasibility and validity of using the SFP in a virtual visit context. The SFP has demonstrated validity for assessing infant stress regulation in controlled laboratory settings (see Mesman et al., 2009), although Moore et al. (2001) provide evidence supporting use of the procedure in home environments. Our findings indicate that not only is it feasible to conduct the SFP using a virtual visit procedure (also see Gustafsson et al., 2021), but that applying a microanalytic coding scheme to video recordings from virtual visits yields data comparable to those collected in a controlled laboratory setting with professional camera capabilities. With respect to feasibility, the percentage of SFP protocols that were completely missing did not differ across lab and virtual visits, although reasons for missingness varied across visit type, with lab visits deemed not codeable largely due to technical issues or infant distress/fatigue (particularly among younger infants), whereas virtual visits were largely missing due to screen distraction among older infants. We note that we quickly adjusted our protocol (i.e., asking mothers to minimize their Zoom screen during the interactive tasks) when it became clear during our initial virtual visits that older infants were distracted by the computer screen. The convenience and scheduling flexibility of virtual visits also enabled us to minimize data loss due to infant distress or fatigue because we could more easily reschedule virtual visits when the mother indicated that another time would be better. These findings are consistent with prior online studies with a live experimenter that show minimal data loss (e.g., Sheskin and Keil, 2018), especially when task and recording/technical procedures have been thoroughly pilot tested and key privacy safeguards (e.g., waiting room, meeting password) are in place (e.g., see Garrisi et al., 2020, for a comprehensive set of recommendations).

Among cases that were deemed codeable, there were consistently higher levels of missing data during the virtual visits, as would be expected. In the laboratory playroom, two professional-grade cameras mounted in opposite corners of the room with zoom, pan, and tilt functions enabled high-quality recordings of mother and infant in split screen format. Nonetheless, despite the much more limited recording capabilities available during the virtual visits (i.e., only one camera angle was available for recording, and there was no ability to pan or tilt the camera to follow the mother and/or infant at moments when they moved out of the camera frame), missing data were relatively minimal and were highest for facial expressions (7–13% missingness, compared with <1–6% missingness for lab visits). Our visit coordinator worked with mothers to obtain the best recording possible, and the relatively low levels of missingness—both in terms of completely missing and partially missing—are acceptable, especially in comparison with levels of missing data observed for asynchronous sessions (e.g., Tran et al., 2017).

With respect to validity, we compared the proportions of behaviors for each infant and maternal code (i.e., facial expressions, vocalizations, and directions of gaze) separately for SFP episodes and time points. Analyses indicated minimal differences in mean proportions. Of the 162 Mann-Whitney *U*-tests conducted for infant behaviors and the 171 tests conducted for maternal behaviors, 4 total were significant at *p* < 0.006 (Bonferroni correction). Further, using a microanalytic coding scheme in which behaviors were coded continuously on a frame-by-frame basis, our assessment of interobserver reliability on 19–25% of visits showed acceptable kappa statistics (>0.65) for all codes at all time points, regardless of visit type. Lastly, at each time point (3, 6, 9 months), infant negative affect, positive affect, and gaze to mother showed the expected patterns of change across play, still and reunion episodes of the SFP as reported in prior work (see Mesman et al., 2009). Importantly, the patterns of infant behavior change were largely consistent across time points, and in no instance did change patterns differ as a function of visit type. In prior studies, maternal and infant behavioral assessments during the SFP have typically been conducted in controlled laboratory settings (e.g., Sravish et al., 2013, but see Moore et al., 2001; Pratt et al., 2015; Busuito and Moore, 2017), raising questions about whether a virtual visit procedure carried out in the familiar home environment (and without researchers physically present) would elicit expected changes in infant affect and behavior. In addressing this concern, our study provides some of the first evidence for the feasibility and validity of assessing mother-infant interaction and infant behavioral regulation during the SFP using a virtual visit format.

Our second objective was to assess infant expressive and receptive communication using the Bayley-III Screening Test. With respect to infant performance, expressive and receptive communication scores at a given time point (3, 6, 9 and 12 months) did not differ as a function of visit type. These results are consistent with prior studies indicating no difference in language performance assessed *via* virtual vs. face-to-face visits among toddlers (Manning et al., 2020) and school-age children (Sutherland et al., 2017). Nonetheless, despite similar performance across the virtual and laboratory visits on the language subtests, there were significantly higher amounts of missing data on the receptive language subtest at the 3-, 6- and 9-month virtual vs. lab visits, which is cause for concern. Although rates of missing data did not significantly differ by visit type at 12 months, there were relatively high rates of missing data for both lab and virtual visits at this time point. The rates of missing data among the expressive subtest scores, in contrast, were relatively low at all time points for both virtual and lab visits.

Unlike the expressive language subtest, which relies mainly on researchers' “incidental observations” of the infant during the course of the virtual or lab visit, the receptive language subtest involves observing the infant's response to administered probes or items. We used stringent scoring criteria, in which subtest scores were considered to be completely missing if one or more items could not be adequately administered and/or scored due to infant compliance, distractions, and/or administrator error. Although we provided mothers with detailed instructions for administering the receptive language items, missing data during virtual visits at 3, 6, and 9 months was mainly due to faulty administration and related difficulty in scoring the infant response. At 12 months, missing data at both virtual and lab visits were predominantly due to infant fatigue, noncompliance, and/or distractions. Taken together, our findings for the Bayley-III Screening communication subtests indicate high levels of feasibility and validity for assessing *expressive language via* a virtual visit procedure in which the infant and mother engage in a variety of interactive tasks that permit observing a range of infant expressive communication skills. Our confidence in virtual assessments of infants' expressive language specifically is corroborated by Manning et al.'s (2020) report on the validity of a virtual assessment of toddlers' language skills obtained during parent-toddler play session using indicators such as observed mean length utterance and number of different words spoken. Given the relatively large proportions of missing data for *receptive language*, however, we have less confidence in the feasibility of assessing this aspect of infant language development *via* a virtual visit procedure.

Our final objective was to assess maternal perspectives of, and preferences for, virtual visits using a brief survey created for the purposes of this study. At each time point, mothers were significantly more likely to rate virtual visits as “easy” compared with “neutral.” Nonetheless, mothers of 12-month-olds were significantly more likely to rate virtual visits as less easy compared with maternal ratings at other infant ages. Mothers of 12-month-olds also showed a preference for in-person visits. Interestingly, although supplementary analyses indicated that prior experience with virtual visits in the context of the current study was not related to infant or maternal behavioral measures (infant or mother SFP behaviors; infant Bayley language scores), mothers of 9-month-olds reported a greater preference for in-person visits when they had not had prior virtual visit experience, whereas mothers of 12-month-olds indicated preference for in-person visits regardless of prior virtual visit experience. A substantial proportion of mothers of older infants (9- and 12-month-olds) also reported that their interactions with their infants were more natural during lab visits compared with virtual visits, although all comparisons on this item were nonsignificant.

Reasons for preferring in-person visits among mothers of older infants centered on distractions that surrounded virtual visits as well as mothers' desire for support by research staff. Given that 9- and 12-month-olds are “on the go” and more attuned to the wider environment, including the device used during the virtual visit, this pattern of maternal responses is perhaps not surprising. Yet, in-person lab visits may pose other challenges for older infants. Whereas it was possible for younger infants to take brief naps or feeding breaks during lab visits, such breaks were less feasible with older infants. As such, older infants may become more fatigued and fussier for some of the same reasons that mothers found virtual visits challenging. Because we did not collect mothers' ratings of relative ease or challenge of their experiences following in-person lab visits, we were unable to directly compare mothers' perceptions of virtual vs. lab visits. We were also unable to compare missingness, reliability, and validity of observational data from the 12-month visits because only data from the SFP sessions have been coded to date. In this light, we cannot make strong recommendations for the use of virtual visits at 12 months. In contrast, data at 3, 6, and 9 months suggest that virtual visits are an acceptable, useful option for capturing infant socioemotional functioning observed during the SFP and infant expressive communication skills assessed *via* the Bayley-III Screening Test.

We note several limitations of the current study. First and foremost, we did not initially set out to assess the feasibility and validity of a virtual visit procedure. Instead, the study objectives in this report emerged as a result of necessary COVID-related restrictions that required us to pivot to a virtual visit protocol. Given the *ad-hoc* nature of the virtual visits, our sample sizes across visit types and time points were unbalanced, although we did conduct virtual visits at all time points to enable assessment of virtual visit feasibility and validity across a range of infant ages. Second, although few differences emerged between virtual and lab visits on our key study measures, COVID-19 posed a clear design confound. This confound was most concerning with respect to mothers of older infants showing a preference for lab vs. virtual visits. Follow-up analyses indicated that such preferences did not covary with when maternal reports were made relative to the COVID shutdown in March 2020. Nonetheless, more direct assessments of families' COVID-related stressors and experiences (e.g., disruptions to child care and work routines, illness, social isolation) are important to consider in relation to mothers' virtual visit perceptions and preferences. Likewise, certain advantages (e.g., scheduling flexibility) and disadvantages (e.g., distractions in the home) of virtual visits may be heightened due to the pandemic and become less salient in a post-pandemic environment.

In addition to limitations specific to COVID-19, the use of virtual visits to assess infant socioemotional and communicative competence requires consideration of broader advantages and disadvantages. Among older infants who are more mobile (crawling, walking), the lack of cameras that could follow the infant and often the lack of contained space in the home increased challenges of conducting assessments of mother-infant interactions with older infants. Further, distractions or interruptions from family members or pets during virtual visits can be a hindrance while carrying out standardized assessments, although we aimed to proactively limit distractions. During visits, mothers were also asked to minimize their Zoom windows during interactive tasks to minimize screen distractions. As already noted above, an important advantage of virtual visits was greater convenience and flexibility in scheduling and/or rescheduling visits as needed due to infant sleep schedule or mood. For instance, we were able to minimize such missing data during our virtual visits by offering mothers the opportunity to schedule a “catch-up” visit if their infant became fussy/tired. Although we provided this option at both lab and virtual visits, mothers were much more likely to schedule a catch-up virtual visit (*n* = 32) vs. a catch-up lab visit (*n* = 15). Not only does such flexibility in resuming virtual visits at a later time when infants are more alert decrease the likelihood of some types of missing data, it may also increase researchers' ability to more accurately capture infants' levels of competence.

We highlight two additional advantages of virtual visits that may provide further motivation for using such procedures to assess infant socioemotional development beyond the COVID-19 pandemic. First, virtual visits may provide a method to more effectively recruit fathers. Although we required mothers' participation for the current study to be consistent with our laboratory procedures, including fathers in virtual visit procedures will likely provide more flexibility to families and thereby increase participation rates. Research on the dynamics of parent-infant interaction and infant development has focused almost exclusively on mothers (e.g., see Davis and Logsdon, 2011), even though fathers have increasingly taken on caregiving roles over the past several decades and make unique contributions to children's socioemotional and cognitive outcomes (e.g., Cabrera et al., 2018; Ruíz et al., 2019). Research indicates, however, that on average, fathers who agree vs. decline to participate in high-commitment, high-stress research procedures (e.g., multiple videotaped procedures) differ on a host of factors, including education, race/ethnicity, infant characteristics, and family functioning (Costigan and Cox, 2001), suggesting the need for research approaches that are less burdensome to families and to fathers, in particular. Changes to family routines and stress on the whole family system brought about by the pandemic (Prime et al., 2020) further underscore the importance of capturing the larger family context and infants' experiences with mothers as well as fathers when present in the home. Virtual visits provide a novel, cost-effective, and family-friendly way to involve fathers more directly in research on infant development and family dynamics.

Second, and in a related vein, virtual visits may provide developmental researchers opportunities to recruit samples with greater racial, ethnic, socioeconomic and/or geographic diversity. To do so, however, researchers will need to be mindful of the “digital divide” faced by participants from rural communities and/or lower SES backgrounds and the obstacles they face in terms of access to reliable computers and internet connectivity (see Lourenco and Tasimi, 2020; van Dijk, 2020). In addition, families characterized by lower socioeconomic status may have less easy access to physical space with few distractions. As such, infant or dyad performance on virtual visit tasks could be impeded due to higher rates of technical issues and/or distractions, and further validation of virtual visit procedures among geographically and socioeconomically diverse samples is warranted.

In sum, the COVID-19 pandemic has accelerated the use of virtual visits by developmental researchers, and past work demonstrating validity of these techniques has predominantly focused on assessments of infant cognition. Our findings indicate that data obtained from assessments of infant socioemotional and language functioning using a synchronous virtual visit procedure are comparable to those obtained during in-person lab visits. Although the use and validation of these new procedures during a global pandemic present inherent limitations, infant assessments conducted *via* Zoom and other remote platforms are likely to be used well beyond the current pandemic. Developmental researchers should continue to assess their feasibility and validity.

## Data Availability Statement

The raw data supporting the conclusions of this article will be made available by the authors, without undue reservation.

## Ethics Statement

The studies involving human participants were reviewed and approved by the Office for the Protection of Research Subjects, University of Illinois at Urbana-Champaign. Written informed consent to participate in this study was provided by the participants' parents.

## Author Contributions

NM contributed to the conception and design of the study. NM, YH, XL, MF, and JCB contributed to the design of the virtual visit procedures. YH, XL, and MF contributed to oversight of data coding and scoring. NM and YH contributed to the statistical analyses and presentation of results in figures. XL, MF, JCB, and JMB contributed to writing sections of the manuscript. All authors contributed to creating tables, revising the manuscript, and approving the submitted version.

## Funding

This research was supported by grants from the National Institute of Mental Health (R21MH112578), the National Institute on Drug Abuse (R34DA050256), and the National Institute of Food and Agriculture, U.S. Department of Agriculture (ILLU-793-368) to the first author.

## Conflict of Interest

The authors declare that the research was conducted in the absence of any commercial or financial relationships that could be construed as a potential conflict of interest.

## Publisher's Note

All claims expressed in this article are solely those of the authors and do not necessarily represent those of their affiliated organizations, or those of the publisher, the editors and the reviewers. Any product that may be evaluated in this article, or claim that may be made by its manufacturer, is not guaranteed or endorsed by the publisher.
